# Inhibiting saccades to a social stimulus: a developmental study

**DOI:** 10.1038/s41598-020-61188-8

**Published:** 2020-03-12

**Authors:** F. Geringswald, A. Afyouni, C. Noblet, M.-H. Grosbras

**Affiliations:** Aix-Marseille Université, CNRS, Laboratoire de Neurosciences Cognitives, Marseille, France

**Keywords:** Saccades, Social behaviour, Human behaviour

## Abstract

Faces are an important source of social signal throughout the lifespan. In adults, they have a prioritized access to the orienting system. Here we investigate when this effect emerges during development. We tested 139 children, early adolescents, adolescents and adults in a mixed pro- and anti-saccades task with faces, cars or noise patterns as visual targets. We observed an improvement in performance until about 15 years of age, replicating studies that used only meaningless stimuli as targets. Also, as previously reported, we observed that adults made more direction errors to faces than abstract patterns and cars. The children showed this effect too with regards to noise patterns but it was not specific since performance for cars and faces did not differ. The adolescents, in contrast, made more errors for faces than for cars but as many errors for noise patterns and faces. In all groups latencies for pro-saccades were faster towards faces. We discuss these findings with regards to the development of executive control in childhood and adolescence and the influence of social stimuli at different ages.

## Introduction

Faces are amongst the most salient visual stimuli that we encounter daily. They convey information paramount for social interactions, such as identity, internal state and intentions. A large corpus of research has demonstrated that faces are processed by specifically tuned mechanisms in the primates’ visual system^1–4^. In addition, faces attract and retain attention faster and more strongly than other objects: independently of low-level visual saliency, even in complex scenes, we look at faces preferentially and more quickly than other objects^5–7^ or animal faces^6,8,9^. We can detect faces more efficiently^10–12^, while is it difficult to ignore them, even if this is detrimental to the task^5,13–15^.

When this prioritized processing emerges in ontogeny and how it develops is unclear. We know that from very early on infants like to look at faces and spend a considerable amount of time doing so^16–19^. The dominant theories propose that this precocious bias for face-like stimuli, coupled with the intrinsic incentive values of faces, would shape the visual and the orienting systems towards becoming increasingly attuned to faces^20^. In accordance, like adults, children and adolescents tend to fixate more faces than other objects when looking at complex visual scenes^21–23^. Yet we also know that many aspects of face processing, such as identity or facial expression recognition, are not fully mature in terms of accuracy and speed, until at least mid-adolescence^24–28^, and that the specialization of brain circuits for faces emerges gradually^29–35^. Some of these findings also suggest a non-linear development with a plateau^36^, or even a dip^37^ in performance at the beginning of adolescence, followed by later additional improvement. This has been interpreted as a consequence of a re-organisation of the face processing system during adolescence to accommodate new demands in social relationships specific of this period, such as forming confiding friendships and romantic relationships^38^. Surprisingly, the development of the special effect of faces for orienting attention beyond infancy, and in particular how this might change during adolescence has remained poorly investigated so far. A few studies have suggested that children and adolescents, compared to adults, have more difficulty inhibiting a response to emotional faces^39^ and are more distracted by them^40^. It is not clear however whether this effect is specific to faces, i.e. a social stimulus, or could occur with any emotional or arousing stimulus^41^. To our knowledge, how orienting and inhibition of responses to non-emotional faces change with development is still unexplored.

This is even more surprising in view of the extensive literature debating putative impaired face processing abilities, including orientation to faces, in neurodevelopmental disorders like autism^42–45^. In this domain, however, most investigations rely on study-based control groups but lack normative developmental comparison data.

**Figure 1:**
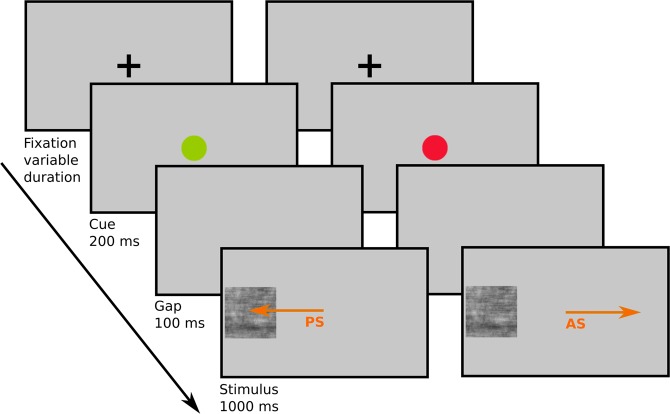
Schematic diagram of an experimental trial and exemplary stimuli. (A) Each trial consisted of a fixation stimulus (variable presentation time), followed by a green or red central cue indicating the saccade task (200 ms), a blank screen (100 ms) and a peripheral target stimulus that was presented either on the left or on the right (1000 ms).

Our goal here was to investigate whether the prevalent effect of faces on the control of spatial orienting changes during late childhood and adolescence. To this end we used the anti-saccade task, with either faces, cars or visual noise patterns as stimuli (see Fig. 1). In this task, participants are asked to either look towards a stimulus that appears in the periphery (pro-saccade) or inhibit this reflexive response and look away from the stimulus (anti-saccade). In adults, pro-saccades to faces are initiated faster than pro-saccades to other stimuli^5,6^. Moreover, the number of anti-saccades direction errors is increased when the stimulus is a face, indicating an interaction between the inhibitory control of involuntary overt orienting and high-level visual processing involving social information^5,13^. The anti-saccades instruction is well understood by even young children^46^ and the task has been used extensively in developmental studies to characterize the maturation of cognitive control. It has demonstrated, as in adults^47^, a good test-retest reliability (ICC 0.84 in a study with 104 participants^47^), making it an instrument of choice to characterize development^48^. Although children from about 5 years old can perform the task, directional errors are frequent and decrease sharply until about 15 years of age and are stabilized by 20^49–58^. This change has been linked to the development of other aspects of cognitive control, such as working memory^59^, and to be largely independent from improvement in visual processing speed^60^. In addition, theoretical and empirical work suggests that, during adolescence, cognitive control is particularly affected in emotional or social contexts, to a greater extent than in other “cold” situations^61,62^. Accordingly, we expected to replicate the decrease in anti-saccades error rates with age, but that the modulation of performance as a function of whether the stimulus has a social connotation or not would vary as a function of age. More specifically, we expected the relative increase in error rates for faces compared to objects or noise stimuli to be even exaggerated in adolescents compared to children or adults. In addition, while biases for looking at faces have been documented from an early age in infants, whether children look at faces faster than at objects is unknown. Due to the immaturity of face processing, we expected the difference in pro-saccades latencies for faces and objects to appear gradually, and possibly non-monotonously, during late childhood and adolescence.

## Results

We tested 139 participants aged 8–22, divided into four age groups based on age and pubertal status: children (age 8–11), early adolescents (age 12–14), adolescents (age 15–17) and young adults (age 18–22). They performed a mixed pro- and anti-saccades task, where the target stimulus was either a face, a car or a noise pattern, all equalized for low-level properties. We first concentrated on our two main hypotheses and thus analysed two parameters that have been shown to be influenced by stimulus category in adults and also to change with age: (i) *antisaccades direction error rate* reflects the ability to inhibit the automatic response and reprogram a voluntary response away from the stimulus. (ii) *pro-saccades latency* reflects the strength of the orienting effect of the stimulus on oculomotor execution. In addition, we report analyses for saccadic parameters that have been shown to change with age, in order to replicate previous developmental studies and to explore whether these factors could also be influenced by stimulus category at different ages. *Antisaccades latency* and *cost on latency* (see below) reflect the difficulty in performing the task; the *proportion of express saccades* and the *intraindividual variability of latencies* reflect the maturation of control over fixation and saccade execution^56^. Lastly saccadic duration or *peak velocity* is thought to be dependant of subcortical systems (brain stem generators or superior colliculus), with changes with age having yielded conflicting results^46,56^. We thus want to add to this corpus of work on developmental changes.

For each of these parameters independently we investigated, using analyses of variance (ANOVAs), the influence of stimulus category across age groups.

In an initial analysis we included the within-subject factor stimulus laterality. As none of the interactions with age group were significant, we pooled data across left and right hemifield trials. Likewise we did not observe any gender effect, and thus we present the analyses without this factor. The final analysis included data from 31 children, 29 young adolescents, 22 adolescents and 31 young adults. Results from statistical tests are summarized on Tables 1 and 2.

**Table 1:** Statistical results of the between-group analyses of image category effect in the anti-saccade task for direction error rate, latencies, latencies for errors, and cost on latencies.

Effect	DFn	DFd	Direction error rate				Latencies correct				Latencies errors				Cost on latencies			
			F	p	η^2^_P_	η^2^_G_	F	p	η^2^_P_	η^2^_G_	F	p	η^2^_P_	η^2^_G_	F	p	η^2^_P_	η^2^_G_
Age group	3	109	27.24	3.2*10^−13^	0.429	0.402	7.97	8.0*10^−5^	0.180	0.171	5.38	0.002	0.131	0.112	2.82	0.042	0.072	0.062
Image	2	218	9.59	1.0*10^−4^	0.081	0.009	0.36	0.699	0.003	<0.001	1.31	0.272	0.012	0.002	6.10	0.003	0.053	0.009
Interaction	6	218	3.31	0.004	0.083	0.009	0.98	0.441	0.026	0.002	0.66	0.686	0.018	0.003	1.11	0.357	0.030	0.005

**Table 2:** Statistical results of the between-group analyses of image category effect in the pro-saccade task for direction error rate, mean latencies, variability of latencies, express saccade rate, and peak velocity.

Effect	DFn	DFd	Direction error rate				Latencies correct				SD latencies correct				Express saccades				Peak Velocity			
			F	p	η^2^_P_	η^2^_G_	F	p	η^2^_P_	η^2^_G_	F	p	η^2^_P_	η^2^_G_	F	p	η^2^_P_	η^2^_G_	F	p	η^2^_P_	η^2^_G_
Age group	3	109	14.01	8.6*10^−8^	0.278	0.200	6.29	6.3*10^−4^	0.148	0.141	19.97	2.2*10^−10^	0.355	0.313	3.35	0.022	0.084	0.081	2.12	0.102	0.055	0.054
Image	2	218	3.69	0.027	0.033	0.012	32.58	4.2*10^−13^	0.230	0.014	7.20	9.4*10^−4^	0.062	0.011	13.18	3.9*10^−6^	0.108	0.005	9.80	8.4*10^−5^	0.083	0.002
Interaction	6	218	1.13	0.344	0.030	0.011	0.46	0.837	0.013	0.001	1.27	0.271	0.034	0.006	0.85	0.532	0.023	0.001	1.04	0.400	0.028	0.001

### Anti-saccades direction error rate

**Figure 2:**
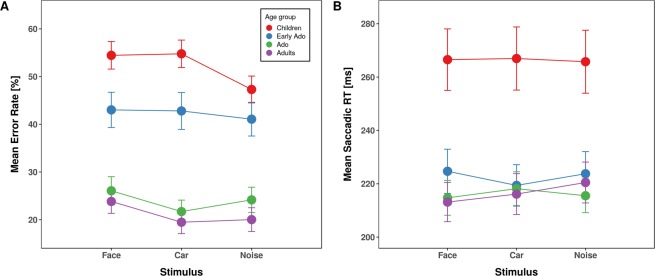
Averaged saccade error rates (left panel) and latencies (right panel) for anti-saccades as a function of stimulus type separated for children (red), early adolescents (blue), adolescents (green) and young adults (purple). Error bars depict the standard error of the mean.

The ANOVA including the between-subjects factor age group and the within-subject factor stimulus type (face, car, noise pattern), yielded a significant main effect of age-group [*F*(3,109) = 27.24, *p* = 3.2*10^−13^, *η²*_*G*_ = 0.402, *η²*_*P*_ = 0.429] (Fig. 2A). Childrens’ overall error rate was significantly elevated compared to all other age groups (children, 52.18%; early adolescents, 42.30%, *p* = 0.030; adolescents, 23.99%, *p* = 1.8*10^−8^; adults, 21.11%, *p* = 6.8*10^−12^) and early adolescents made significantly more errors than adolescents (*p* = 2.7*10^−4^) and adults (*p* = 1.3*10^−6^) while error rates between adolescents and adults were comparable (*p* = 0.364). The main effect of stimulus type was also significant [*F*(2,218) = 9.59, *p* = 1.0*10^−4^, *η²*_*G*_ = 0.081, *η²*_*P*_ = 0.429], reflecting overall higher error rates for faces compared to noise patterns (37.59 vs. 33.72%, *p* < 0.001) and to cars (35.58%, *p* = 0.015). The difference in error rates for cars and noise patterns was not significant (*p* = 0.111).

Importantly, the interaction between age group and stimulus type was significant [*F*(6,218) = 3.31, *p* = 0.004, *η²*_*G*_ = 0.009, *η²*_*P*_ = 0.083]. To explore this interaction, we first compared the mean error rates for each stimulus type against each other within each age group. Children made significantly more errors when they had to make a saccade away from a face (54.46%, *p* = 0.003) or a car stimulus (54.78%, *p* = 0.004) compared to a noise pattern (47.30%), while error rates between face and car stimuli did not differ (p = 0.833). In early adolescents there was no difference between stimuli (mean error rates 43.02, 42.80, and 41.08% for face, car, and noise stimulus respectively, all *p*s > 0.568). Adolescents made more errors when saccading away from a face compared to a car (26.06 and 21.70% errors respectively, *p* = 0.001) but none of the comparisons against noise pattern stimuli was significant (24.17% errors, all *p*s > 0.201). In contrast to the other age groups, adults made more errors when saccading away from a face compared to a noise pattern stimulus (23.83 and 20.02% errors respectively, *p* = 0.026) as well as in comparison to a car (19.47% *p* = 0.021). Error rates were not significantly different between car and noise stimuli (*p* = 0.747). The conclusions of these results remained unchanged when we analysed the data including the 14 participants who had been excluded due to performance more than 2 SD away from the mean of their group (See Methods and Supplementary information).

**Figure 3:**
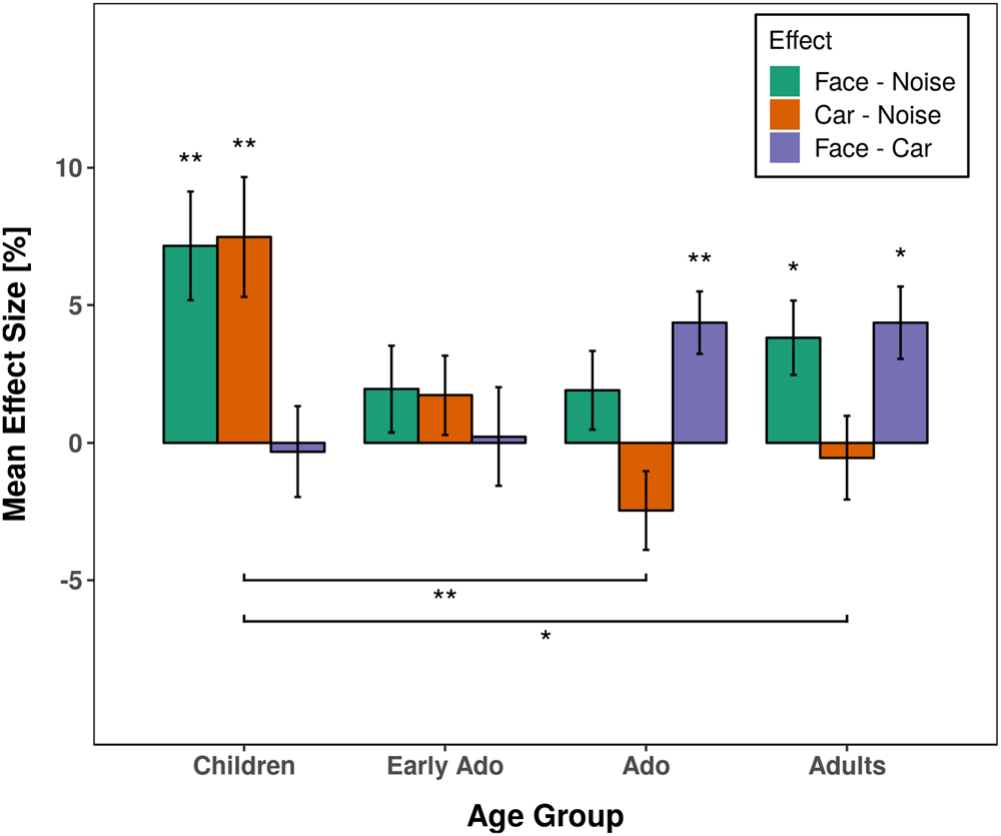
Averaged difference in saccade error rates between stimulus types for anti-saccades as a function of age group. Error bars depict the standard error of the mean. Asterisks depict statistically significant differences between stimulus effects within and between experimental groups. *p < 0.05, **p < 0.01.

To represent and compare the effect of stimulus type on anti-saccade errors between age groups more specifically, we subsequently analyzed the difference between error rates for faces versus noise (face effect) and cars versus noise (car effect) with post-hoc t-tests (see Fig. 3). The face effect was numerically elevated for children (7.16%) compared to early adolescents 1.95%, adolescents 1.90%, and young adults 3.81% but none of the inter-group comparisons was statistically significant (all *p*s > 0.270). The car effect was also elevated in children (7.48%) which was statistically more than in adolescents (−2.46%, *p* = 0.002) and adults (−0.55%, *p* = 0.012), but not significantly different from early adolescents (1.72%, *p* = 0.083). None of the other comparisons between age groups approached significance (all *p*s > 0.446). The difference between face and car was numerically elevated for adults (4.36%) and adolescents (4.36%) compared to children (−0.32%) and early adolescents (0.22%) but none of these differences reached significance in the between-groups comparison (all *p*s > 0.160).

### Anti-saccades latencies

We analyzed response times only for correct anti-saccades (Fig. 2B). The ANOVA revealed a significant main effect of age group [*F*(3,109) = 7.97, *p* = 8.0*10^−5^, *η²*_*G*_ = 0.171, *η²*_*P*_ = 0.180] due to overall slower anti-saccades in children (266 ms) compared to all other age groups (early adolescents, 222.6 ms; adolescents, 216.1 ms; young adults, 216.6 ms; all *p*s < 0.002). Reaction times were comparable between the other age groups (all *p*s = 1). Neither the main effect of Stimulus type [*F*(2,218) = 0.36, *p* = 0.700, *η²*_*G*_ < 0.001, *η²*_*P*_ = 0.003] nor the interaction were significant [*F*(6,218) = 0.98, *p* = 0.441, *η²*_*G*_ < 0.002, *η²*_*P*_ = 0.026].

### Costs on latencies

Previous studies have reported that the difference in reaction times between anti-saccades and pro-saccades, sometimes called “anti-saccades cost”^63^ or “anti-saccades effect”, which reflect difficulty in inhibiting the automatic saccade, decreases with age following an inverse function^56^. We thus analysed the anti-saccade cost by calculating the difference between mean response times for anti-saccades and mean response times for pro-saccades. We inquired whether this metric could differ across stimulus types and age groups.

The main effect of age group was significant [*F*(3,109) = 2.82, *p* = 0.042, *η²*_*G*_ = 0.062, *η²*_*P*_ = 0.072]. Children exhibited higher anti-saccade costs (67.03 ms) compared to early adolescents (52.27 ms), adolescents (45.19 ms) and adults (47.38 ms). The main effect of stimulus type was also significant [*F*(2,218) = 6.10, *p* = 0.003, *η²*_*G*_ = 0.009, *η²*_*P*_ = 0.053]. Anti-saccade reaction time costs were higher for face stimuli (56.70 ms) compared to noise pattern stimuli (49.34 ms, *p* = 0.007) and for car stimuli (54.76 ms) compared to noise patterns (*p* = 0.017). Face and car stimuli were not significantly different (*p* = 0.385). The interaction between stimulus and age was not significant [*F*(6,218) = 1.11 *p* = 0.357 *η²*_*G*_ < 0.005, *η²*_*P*_ = 0.030].

### Relationship between errors and latencies

**Figure 4:**
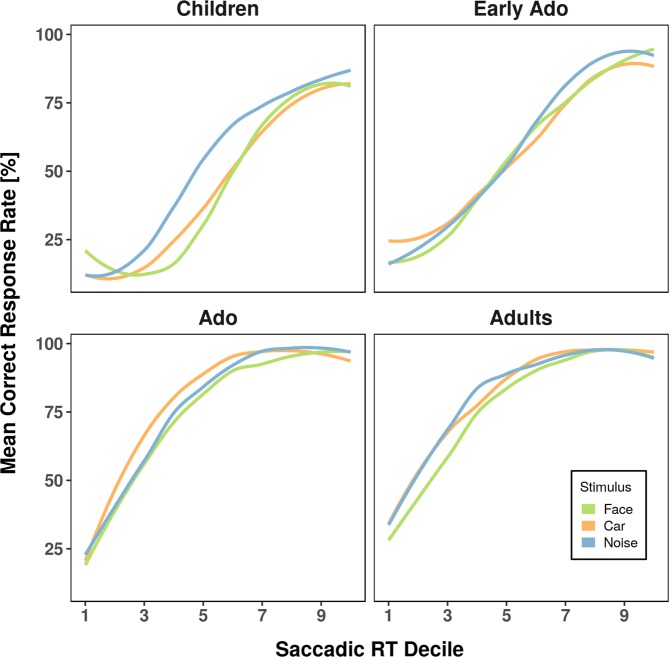
Averaged conditional accuracy functions for anti-saccades separated for face (green), car (orange), and noise (blue) stimulus types.

To explore qualitatively whether errors were linked to reaction times, we computed the percentage of errors for each reaction time decile for each individual and each stimulus type (Fig. 4)^64,65^. We averaged these conditional accuracy functions within each age group. Figure 4 shows that the increased error rate for face and car stimuli, when present, concerns most of the response-times distribution. The increased error rate to these stimuli is thus not driven by impulsive responses, in which case the difference between stimuli would primarily appear for the shortest latencies.

### Latencies for anti-saccades errors

The latencies for the erroneous pro-saccades in the anti-saccades trials showed a main effect of age group [F(3,107) = 5.38, *p* = 0.002, *η²*_*G*_ = 0.112, *η²*_*P*_ = 0.131], with longer response times for children as compared to all the other groups (mean 171 ms vs 144 ms, 143 ms and 142 ms, for the children, early adolescents, adolescents and adults respectively). Nevertheless there was no effect of the stimulus category [F(2,214) = 1.31, *p* = 0.272, *η²*_*G*_ = 0.002, *η²*_*P*_ = 0.012], nor interaction [F(6,214) = 0.66, *p* = 0.686, *η²*_*G*_ = 0.003, *η²*_*P*_ = 0.018].

### Pro-saccades errors

**Figure 5:**
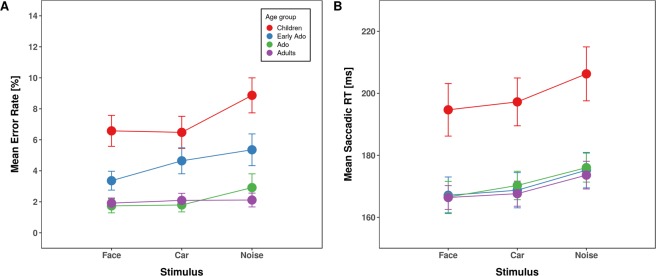
Averaged saccade error rates (left panel) and reaction times (right panel) for pro-saccades as a function of stimulus type separated for children (red), early adolescents (blue), adolescents (green) and young adults (purple). Error bars depict the standard error of the mean.

The ANOVA, including the between-subjects factor age-group and the within-subject factor stimulus type (face, car, noise pattern), revealed a significant main effect of age group [*F*(3,109) = 14.01, *p* = 8.6*10^−8^, *η²*_*G*_ = 0.200, *η²*_*P*_ = 0.278] due to overall increased error rates in children (7.31%) compared to the other age-groups (early adolescents, 4.46%; adolescents, 2.15%, young adults, 2.04%; all *p*s < 0.021) and increased error rates of early adolescents compared to adolescents and adults (all *p*s < 0.021) Fig. 5A. In addition, the main effect of stimulus type was significant [*F*(2,218) = 3.69, *p* = 0.027, *η²*_*G*_ = 0.012, *η²*_*P*_ = 0.033]. Error rates were significantly reduced for face stimuli (3.53%) compared to noise pattern stimuli (4.96%, *p* = 0.035). None of the comparisons with car stimuli (3.89%) reached significance (all *p*s > 0.125). The interaction with age was not significant [*F*(6,218) = 1.13, *p* = 0.344, *η²*_*G*_ < 0.011, *η²*_*P*_ = 0.030].

### Pro-saccades latencies

We observed a significant main effect of age-group [*F*(3,109) = 6.29, *p* = 6.3*10^−4^, *η²*_*G*_ = 0.141, *η²*_*P*_ = 0.148] Fig. 5B. Overall, childrens’ saccadic reaction times were significantly slower (199 ms) compared to the other age groups (early adolescents, 170 ms; adolescents, 171 ms; and adults, 169 ms; all *p*s < 0.008). Saccadic response times did not differ significantly between the other age-groups (all *p*s = 1). The main effect of stimulus type was also significant [*F*(2,218) = 32.58, *p* = 4.2*10^−13^, *η²*_*G*_ = 0.014, *η²*_*P*_ = 0.230], reflecting overall significantly faster pro-saccades to faces (174 ms) compared to noise patterns (183 ms, *p* = 1.5*10^−13^) as well as faster pro-saccades to cars (177 ms) compared to noise patterns (*p* = 7.4*10^−7^). The difference in reaction times between faces and cars just missed significance (*p* = 0.053). The interaction between age-group and stimulus type was not significant [*F*(6,218) = 0.46, *p* = 0.837, *η²*_*G*_ < 0.001, *η²*_*P*_ = 0.013], indicating comparable effects of stimulus type on reaction time for all age-groups.

### Intra-individual variability in pro-saccades latencies

ANOVA of the standard deviation of saccades latency showed a significant decrease with age (children: 83.4 ms; early adolescents: 56.2 ms; adolescents: 42.3 ms; adults: 39.9 ms) [F(3,109) = 19.97, *p* = 2.2*10^−10^, *η²*_*G*_ = 0.313 *η²*_*P*_ = 0.355]. A significant main effect of stimulus type [F(2,218) = 7.20, *p* = 9.4*10^−4^, *η²*_*G*_ = 0.01, *η²*_*P*_ = 0.062] was due to greater response time variability for noise pattern (60.3 ms) than for faces (53.6 ms, *p* = 2.2*10^−4^) and cars (55.5 ms, *p* = 0.030). There was, however, no significant interaction between age group and stimulus type [F(6.218) = 1.27, *p* = 0.271, *η²*_*G*_ = 0.006, *η²*_*P*_ = 0.034].

### Percentages of express saccades

Saccades with latencies between 80 and 130 ms are typically classified as express saccades^66^, which can be distinguished from regular saccades in terms of their neural substrates (rev. in^67^). The proportion of express saccades is thought to reflect the state of attentional engagement^54^. We analysed the proportion of express saccades across age-groups and conditions (only correct pro-saccades, with arc-sin root transformation to comply with non normality). The ANOVA revealed a main effect of age-group [F(3,109) = 3.35, *p* = 0.022, *η²*_*G*_ = 0.081, *η²*_*P*_ = 0.084], reflecting increased proportion of express saccades in the early adolescents (34.9%), compared to young adults (19.9%, *p* = 0.019), but not statistically different from children (26%) or the oldest adolescents (22.4%, all *p*s > 0.117). The effect of stimulus was also significant [F(2,218) = 13.18, *p* = 3.9*10^−6^, *η²*_*G*_ = 0.005, *η²*_*P*_ = 0.108] reflecting higher proportion of express saccades for both faces (26.6%) and cars (26.9%) compared to noise pattern stimuli (24.2%, all *p*s < 1.8*10^−4^). There was no interaction between age-group and stimulus type [F(6,218) = 0.85, *p* = 0.532, *η²*_*G*_ < 0.001, *η²*_*P*_ = 0.023].

### Peak velocity for pro-saccades

There was no change in peak velocity with age [F(3,109) = 2.12, *p* = 0.102, *η²*_*G*_ = 0.054, *η²*_*P*_ = 0.055], but a main effect of stimulus category [F(2, 218) = 9.80, *p* = 8.4*10^−5^, *η²*_*G*_ = 0.002, *η²*_*P*_ = 0.083], reflecting slightly faster saccades for faces (415.9°/s) and cars (413.4°/s) compared to noise patterns (409.6°/s, all *p*s < 0.008). We observed no significant interaction between age and stimulus [F(6,218) = 1.04, p = 0.400, *η²*_*G*_ < 0.001, *η²*_*P*_ = 0.028].

## Discussion

We aimed to investigate the developmental landmarks of the prioritization of faces in an oculomotor task that requires inhibitory control. We compared performance in pro or antisaccades task using different categories of stimuli in age groups corresponding to biological and social developmental landmarks: children before puberty and entry into secondary school; early adolescents during puberty when executive control and social perception are still not mature, a period which corresponds also to important changes in social environment with the start of secondary school (middle school in France); adolescents after puberty when executive control is adult-like but social perception is still refining (high school period in France), and adults. As expected, we observe that task performance improves with age between childhood, different stages of adolescence and adulthood: this is reflected by a significant decrease in anti-saccade errors as well as faster and less variable reaction times for pro- and anti-saccades. We also replicate, in adults, the higher anti-saccades error rate when the stimulus is a face compared to cars or meaningless noise stimuli, indicating the additional demand to suppress the automatic orienting response to faces. Crucially this pattern varies with age, but contrary to our expectation this change is not characterized by higher attraction to faces in adolescence. Children show the face-effect but it is also apparent for cars, while early adolescents show the same error rate for all stimuli categories. Older adolescents commit more errors for faces as compared to cars, but not significantly more than in the noise patterns condition. With respect to reaction times participants from all age-groups are faster to look towards faces, with no evident developmental changes. We discuss these results with regards to the maturation of the interaction between face processing and executive control.

### General task performance improves with age

Correct anti-saccades require inhibiting the reflexive movement towards the stimulus appearing on the screen and reprogramming a voluntary movement towards the opposite side of the screen^56^. This capacity improves with age, likely due to the development of inhibitory control and in relation to maturation of cortico-striatal networks^58,68^. Our results show improvement from childhood until late adolescence, with no difference between the older adolescents group and the adults group. They replicate those from previous studies that used meaningless visual targets, such as dots or stars, and showed that the mean error rate increases until about 15 years of age^46,49,56,57,69^. Also concordant with previous findings, our results show faster reaction times for both pro- and anti-saccades with age, with a stabilization around age 12, as well as decreased variability with age^56,57,70–72^. Interestingly the reduction of latencies with age was steeper for anti-saccades, which effectively resulted in a reduction of anti-saccade cost with age, which also replicates previous finding^56^. This indicates that an improvement of executive control in the context of competing spatial stimuli occurs on top of improvement of control oculomotor command.

In addition to making the most anti-saccade errors, children also made pro-saccade errors. This could be due to difficulty switching tasks. Although our data do not allow us investigating precisely the effect of alternating or not a pro- and an anti-saccade trial, due to insufficient numbers of trials and imbalanced sequences after removing noisy trials, others have reported that the cost of switching task, represented as the increased reaction time when a trial is of a different task condition as the preceding trial, decreases across childhood until young adulthood^73^. Concordant with this hypothesis, previous reports of developmental changes in performance in pro- and anti-saccades using blocked designs did not report any age effect on pro-saccade errors^56,69^.

Next, we asked whether this immature executive control was affected by the category of visual stimuli, in particular whether they are social or not, in the same manner as it has been described in adults. More specifically we investigated whether faces had the same influence on the orienting system across development.

### The effect of stimulus on task performance changes with age

Our data replicate the face-effect on anti-saccade errors observed in the study by Morand and colleagues *et al*. (2010)^5^: adults make more anti-saccade errors towards faces than towards cars and noise stimuli. This indicates that faces attract involuntary attention more than other visual stimuli, whether they are high level (cars) or low level (noise), therefore suggesting an interaction between high-level visual processing and involuntary orienting. This feature was expressed differently as function of age, however. Children (age 8–11) made more errors to faces than to noise, but displayed also a similarly high error rate for cars. The early adolescents (age 12–14) made the same number of errors for all stimulus categories. The older adolescents (age 15–17), whose global error rate did not differ from adults, made more errors for faces than for cars, but displayed a high number of errors for noise patterns. Altogether this indicates that the categorical effect of visual processing on inhibitory control, reflected by increased attraction to faces compared to cars, arises in late adolescence. This is in line with neuroimaging studies showing that the category-specificity of visual ventral stream face-sensitive regions in the brain (that is their higher activity in response to faces as compared to any other categories) is not prominent in children and emerges gradually during adolescence^32,33,74–76^. Also, the functional integration of this network, in particular of occipitotemporal regions, is remodelled from childhood to adolescence^77^, which presumably could impact the relationship with brain networks involved in executive control. This contrasts with the cortical activity engaged for manufactured objects which seems stable from childhood to adulthood^78,79^. The fact that there was no interaction between stimulus category and age-related changes in latencies and peak velocities, for both pro-saccades and anti-saccades, with faster responses to faces across age groups, suggests that faces have the same effect on the regulation of the oculomotor command across development. Likewise the absence of interaction between age and stimulus category with regards to the proportion of express saccades, and the fact that errors occurred in most parts of the reaction time distribution, rule out an effect directly on subcortical mechanisms. Indeed, midbrain structures, and in particular the superior colliculus, are prominently involved in the production of these very short latencies saccades, integrating visual and motor information^80^. In addition, converging evidence from neuropsychology and neuropsychology show responses related to face detection in the superior colliculus^81^. If the effect of visual category on oculomotor programing was mainly taking place at the level of superior colliculus we would expect an effect of stimulus category mainly on short latencies and express saccades. Here, what is most likely to change with age is the ability to inhibit the slight advantage for responding to faces compared to other stimuli and to reprogram a response away from this attractive stimulus. These so-called high level processes are thought to rely on a set of cortical brain regions. Thus, the difference in anti-saccades to faces and cars across development might be related to differences in the functional integration between visual areas related to processing faces and cortical regions involved in cognitive control. If so, the observed changes could be related to the way these circuits process faces or the way they process cars or both. This should be tested by a proper neuroimaging study.

Nonetheless, these results can also be explained in terms of motivational salience. The intrinsic value (motivation or emotion) of visual targets has been shown to influence voluntary saccades and anti-saccades errors^82,83^. In a visual search task it has been shown that children were distracted by faces and trains in the same way^84^. So, it is possible that in children, cars, or more generally vehicles, have the same motivational salience than faces. Indeed, in this age range, vehicles are still popular toys that are used in pretend play with a strong personification (for instance evident in the popular cartoons Cars or Thomas the tank engine). This could also contribute to similar activity and connectivity in the occipito-temporal face-selective cortex for faces and cars^85^ and thus potentiality to similar influence on oculomotor control.

In early adolescents, the meaningless noise patterns had the same effect as the cars and faces and in older adolescents, they elicited more errors than cars. The privileged access of faces on orienting is thus on par with that for noise patterns in adolescence. This can also be explained in terms of motivational salience: adolescents might be more attracted by and deploy more effort to interpret these abstracts stimuli. The higher variability in reaction times for these stimuli compared to cars and faces is also consistent with this interpretation, as different individuals might be differently influenced by each of these meaningless stimuli. This is reminiscent of data in other domains showing that adolescents react differently to ambiguous stimuli as compared to children and adults^86,87^. Again, this should be investigated in dedicated studies.

### Limitations and future directions

In order to ascertain that any stimulus effect was not due to low-level visual properties we have manipulated the images to remove colour and equate global amplitude spectra. This results in impoverished, somewhat unrealistic stimuli. Although even children are familiar with seeing grey-scaled images of various resolutions, it is still possible that the perceptual effects resulting from image manipulation affects children, adolescents and adults differently. It would be interesting to test, in future studies, whether more realistic stimuli or stimuli with different attractiveness or emotional value would yield the same distinction, or absence of distinction in the case of early adolescents, between faces and non-face stimuli. This would imply testing more visual categories. This could also allow to disentangle to what extent the developmental pattern we observe, with regards to stimulus effect on performance, may be due to specific changes in the way that cars or faces are processed.

In the same vein, it would be interesting to test whether similar differences could be observed between social and non-social stimuli in other tasks that involve cognitive control but that do not have a direct mapping between visual stimulation and response, as it is the case with oculomotor tasks^60^.

In conclusion, we show that faces are looked at faster than other stimuli already in children, but that they become special compared to other objects for inhibitory control only during adolescence. This offers normative data to evaluate abnormal interaction between social processing and executive control in abnormal neurodevelopmental conditions such as autism or conduct disorder.

## Methods

### Participants

One hundred and thirty-nine volunteers aged 8 to 22 years took part in the study. We assigned participants to four age groups: (1) ‘children’ from 8 to 11 years (end of Primary school in France; mean = 9 years; n = 38; 23 females; 2 left handed); (2) ‘young adolescents’ from 12 to 14 years (Middle school; mean age 13 years; n = 34, 18 females, 2 left handed); (3) ‘adolescents’ from 15 to 17 years (High school; mean age 16 years; n = 25; 17 females; 3 left handed); (4) ‘young adults’ from 18 years to 22 (mean age 20; 20 females, 6 left handed).

Handedness was self-reported as well as information about family and lifestyle including, number of siblings, practice of sport and music, time spent on video-games. In addition, children and adolescents completed the Puberty Developmental Scale to assess their physical development. This is an eight-items gender specific questionnaire targeting changes in secondary sexual characters. For the youngest participants it was filled with the parents. We used the method by Crockett^88^ to convert the PDS scores into Tanner stages, 1: pre-pubertal, 2: beginning puberty, 3: mid-puberty, 4: advanced puberty, 5: post-pubertal). The median tanner stages were 2 in children (SD = 0.9), 3 in young adolescents (SD = 0.9), and 4 in adolescents (SD = 1.0). A Kruskal-Wallis rank sum test indicated that the Tanner stages differed significantly between age groups (χ^2^(2) = 51.77, p = 5.7*10^−12^). Follow-up Dunn’s multiple comparison test^89^ that were corrected according to Holm^90^ were significant for all age-group comparisons (all absolute z > 2.65, p < 0.008), indicating that the four age-groups differed significantly with respect to pubertal development. To test whether pubertal development differed between male and female participants, we performed additional Kruskal-Wallis rank sum test for each age group and corrected for multiple comparisons according to Holm. None of the comparisons reached significance, indicating that pubertal development was comparable between males and females within each age group (all χ^2^(1) < 3.63, *p*s > 0.169).

All participants reported normal or corrected to normal vision and no diagnostic of neurodevelopmental disorder. Participants were not informed about the purpose of the study until they had completed the experiment. The procedures followed the tenets of the Declaration of Helsinki and the study was approved by the Ethics Committee “ Sud Mediterranée 1”. Informed written consent was obtained prior to the experiment from participants and, in the case of minor participants, from their parents in addition. Participants received a voucher compensation of €10 after completing the experiment.

### Apparatus

Stimulus presentation was controlled using E-Prime (version 2.0.1.127, Pychology Software Tools, Pittsburgh, PA, USA) on a portable computer with a 22 inch- LCD monitor (HP Compaq LA2205wg). The monitor was 474 mm (1280 pixels) high and 296 mm (768 pixels) wide and the refresh rate was 60 Hz. The stimuli were viewed binocularly from a distance of 68 cm, leading to a pixel size of approximately 0.03°. The eye position of the left eye was recorded using an Eyelink 1000 Plus Desktop Mount (SR Research Ltd., Mississauga, Ontario, Canada) system, using corneal reflection and pupil tracking. The temporal resolution of the eye tracker was 2000 Hz. Head movements were minimized by stabilizing participants’ head using a chin- and forehead rest. Participants were tested individually in a dimly lit, sound-attenuated chamber.

### Stimuli

Stimuli were the same as used in previous studies by Morand and colleagues^5,91^. Social stimuli included 6 female and 6 male neutral Western Caucasian faces. Object stimuli were constituted of 12 different car models. Noise patterns were 12 different phase-scrambled stimuli that were comparable to the other stimuli with respect to basic visual properties but did not contain any meaningful pattern. To equalize global visual properties, all stimuli were greyscale and normalized for mean amplitude spectrum, luminance and root mean square contrast and were presented on a uniform grey background (for more details, see 5). Stimuli were 5.3° of visual angle wide and 5.5° of visual angle high and were centered 14.5° of visual angle either to the left or to the right from the screen center on the horizontal meridian. A green disk or a red disk with a diameter of 1.2° of visual angle was used as a cue to indicate whether the saccade was to be performed towards the peripheral stimulus or into the opposite direction. The fixation cross was composed of two black line segments subtending 1° of visual angle.

### Task and procedure

Each session started with a 9-point gaze-calibration, followed by a short training comprising 20 trials to familiarize participants with the task Fig. 1. Each trial started with the presentation of the fixation cross that served for manual drift correction. Participants were asked to fixate the cross and the experimenter manually adjusted the calibration for small drifts by accepting a valid fixation that fell within a radius of 2° of visual angle of the screen center via a key press. Immediately after drift correction had been completed, a cue colored either green or red instructing which the saccade task to perform was presented for 200 ms. The cue was followed by the presentation of the background screen for 100 ms, introducing a gap before target stimulus onset; this effectively speeds up saccadic reaction times globally and increases anti-saccade error rates specifically^56^. After the gap, the target stimulus was presented peripherally for 1000 ms and participants either had to saccade to the stimulus (prosaccade in case of green cue) or into the opposite direction (antisaccade in case of red cue). Participants were asked to perform the saccade as fast as possible after the appearance of the central cue. No feedback about the correctness of the response was given.

There were thus 12 trial types: 2 saccade conditions (pro- and anti-saccades) × 3 stimuli categories (face, car, noise pattern) × 2 hemifields (left, right). The actual experiment included six runs containing 84 trials each (seven per condition). Between runs, participants were allowed self-determined breaks. One experimental session lasted approximately one hour.

### Data analysis and data exclusion

Data pre-processing and statistical tests were carried out using R (version 3.3.1^92^. Analyses of variance (ANOVAs) were performed using Type III sums of squares. For all statistical tests, the significance level was set to 0.05. Gaze parameters were identified with the Eyelink Dataviewer Software (SR Research Ltd., Mississauga, Ontario, Canada), using velocity and acceleration thresholds of 35°/s and 9500°/s^2^ respectively for saccade detection. For each trial, the first saccade after stimulus onset was analyzed.

Trials were discarded if (1) the saccade latency was shorter than 80 ms as these were likely to reflect anticipatory responses^93^, (2) the saccade amplitude was smaller than 2° of visual angle, (3) the saccade direction was classified as ‘up’ or ‘down’, (4) any signal loss, for example due to blinks, occurred before the end of the saccade, or if (5) the distance between the saccade start point to the screen center exceeded 1° of visual angle.

Following this procedure, we excluded participants for whom less than 10 valid trials remained in one of the cells of the saccade type (pro- and anti-saccades) X stimulus type (face, car, and noise pattern) X correct saccade direction (left and right visual field) factor combinations. This lead to the exclusion of 12 participants altogether. One participant was excluded in the children group, three participants in the young adolescents group, one participant in the adolescents group, and seven participants in the young adults group, respectively. For the remaining participants, 26.7% of all trials were excluded because they contained saccades with latencies below 80 ms (5.21% of all trials), amplitudes smaller than 2° of visual angle (1.97%), or directions classified as ‘up’ or ‘down’ (0.75%), because they contained periods of signal loss before saccade end (3.93%), or because the saccade start was further than 1° of visual angle from the screen center (13.38%). On average, 369 trials per subject contained a valid saccade. This procedure of data cleaning is standard in oculomotor studies and is critical in developmental studies^94^. This data exclusion rate is similar to other developmental studies^50^.

On the remaining trials we computed, separately for pro- and anti-saccades, the mean reaction time and the error rate defined as the number of directional errors relative to the number of valid trials. To identify participants whose performance was atypical from their respective age group, we defined separate performance criteria for saccade error rates and reaction times. We calculated thresholds (mean plus two times the standard deviation) for each age group and task type (pro- and anti-saccades) and excluded participants whose individual performance exceeded either of these thresholds. Thresholds were based on arcsine square root-transformed mean error rates and log-transformed mean reaction times. In the children group, six participants were removed following this procedure (two participants exceeded the error rate threshold for anti-saccades committing 86.99 and 87.68% of errors each, two participants exceeded the error rate threshold for pro-saccades committing 26.63 and 34.38% of errors each, and two participants exceeded the reaction time threshold for pro-saccades with an average reaction time of 359 and 406 ms each). In the young adolescents group, two participants were removed (one exceeded the error rate threshold for pro-saccades committing 16.14% of errors and one the reaction time threshold for pro- as well as anti-saccades with average reaction times of 419 and 335 ms, respectively). In the adolescents group, two participants were removed (one exceeded the error rate threshold for pro-saccades committing 9.91% of errors and one the reaction time threshold for pro-saccades with an average reaction time of 308 ms). In the young adults group, four participants were removed (one exceeded the error rate threshold for anti-saccades committing 60.00% of errors and one the error rate threshold for pro-saccades committing 8.24% of errors and two the reaction time threshold for pro-saccades with average reaction times of 233 and 234 ms each).

Thus, the data from 31 children, 29 young adolescents, 22 adolescents and 31 young adults remained in the final analysis. There was no group-difference in the number of trials in this final sample: 358 for children, 379 for early adolescents, 383 for the adolescents and 366 for the adults. Also, the average number of trials included in each cell of the factorial design did not differ across conditions nor across groups, as shown in Table 3.

**Table 3:** Number (mean and standard deviation) of analyzed trials for each cell of the experimental design.

		Anti-saccades		Pro-saccades	
		Left	Right	Left	Right
Children	Car	29.68 (8.45)	29.61 (8.52)	31.26 (7.47)	31.00 (7.74)
	Face	28.55 (8.35)	28.03 (8.57)	31.42 (7.95)	30.68 (7.57)
	Noise	29.52 (8.37)	27.74 (8.64)	30.19 (8.13)	30.42 (8.59)
Early adolescents	Car	30.72 (7.29)	30.72 (7.42)	32.72 (6.93)	33.59 (7.24)
	Face	30.66 (6.89)	30.66 (6.68)	33.38 (6.16)	32.93 (6.23)
	Noise	29.93 (7.70)	29.83 (7.27)	32.14 (7.12)	32.14 (6.22)
Adolescents	Car	30.68 (8.91)	32.32 (7.74)	34.41 (7.14)	32.82 (8.35)
	Face	30.14 (8.54)	30.55 (8.54)	33.95 (7.37)	32.59 (8.55)
	Noise	30.32 (8.95)	30.14 (9.37)	33.64 (7.07)	31.64 (8.61)
Young adults	Car	30.61 (8.98)	28.94 (7.78)	32.39 (7.48)	31.55 (7.89)
	Face	29.45 (8.41)	29.58 (8.03)	32.19 (7.43)	31.00 (7.27)
	Noise	29.48 (8.30)	29.16 (7.96)	31.29 (6.38)	30.61 (7.70)

We used mixed-design ANOVA to investigate the effect of age-group (children, early adolescents, adolescents, young adults) and stimulus type (face, car, noise pattern) and the interaction of these two factors on the following dependent variables: Anti-saccades Error Rate; Antisaccade Reaction Times on correct trials; Pro-saccades error rate; Pro saccades Reaction times; Pro-saccades reaction time standard deviation; Anti-saccade cost (or “anti-saccades effect), defined as the difference between the mean reaction time in anti- and pro-saccades conditions^56^. Additional analyses were performed to investigate the effect of gender and target laterality on the age and stimulus effects and their interaction. As these did not bring any significant observation with respect to interaction with age effects, we don’t present them. Post-hoc *T* tests were corrected according to Holm procedure.

The dataset generated during the current study is available from the corresponding author upon reasonable request.

## Supplementary information

Supplementary Information 2.
