# Supplementary material for: Inhibiting saccades to a social stimulus: a developmental study
Source: Sci Rep. 2020 Mar 12;10:4615. doi: 10.1038/s41598-020-61188-8 (PMC7067843; doi:10.1038/s41598-020-61188-8)
Supplement: Supplementary file 1 — Supplementary Information 2. [file 41598_2020_61188_MOESM1_ESM.docx]

**SUPPLEMENTARY INFORMATION**

**Inhibiting saccades to a social stimulus: a developmental study**

Geringswald F.; Afyouni A.; Noblet C.; Grosbras M.-H.*

For the data published in the main manuscript we have exclude 26 of the participants out of the 139 who were enrolled in the study. This was due to poor eye tracking data quality for 12 of them, while 14 were excluded because their performance was more than 2 standard deviation (SD) from the mean of their age group.

The primary reason for excluding participants with relatively strict threshold was to make groups more homogeneous in terms of global performance in the task. In this way, an early-adolescent who would perform worse than her age-mates and more towards the level of children with respect to direction errors rate, for instance, would not be considered in the analysis, in which the main question addresses the interaction between stimulus category and performance. To clarify, while we do not expect all participants of a particular age group to behave in the same way, we arranged for the general performance in the task to be bounded within each group, before testing the additional factor of stimulus category within each age group.

Nevertheless, some valuable data might be overlooked that way. Therefore, we have also analysed the data including also the participants that had been considered outliers with regards to general task performance within their age-group. The main conclusions are not impacted by this analysis. Notably, the interaction of age group and image category on anti-saccade errors remains significant [F(6,246) = 3.40, p = .003], characterized by i) increased error rates to faces compared to noise for children (p = .0003) and adults (p = .015), but not young adolescents (p = .344) and adolescents (p = .192), ii) the effect specific to cars versus noise being significant for children only (p = .001; all other age groups p > .19), and iii) the effect face versus car being significant for adolescents (p = .001) and adults (p = .024= but not children (p = .837) or young adolescents (p = .712). None of the other measures concerning anti-saccades showed a significant age-group by stimulus category interaction (all Fs < 1.07, ps > .384) and neither was any of the interactions concerning pro-saccades significant (pro-saccade error rate F = 1.947, p = .074; all other Fs < .897, ps > .498).

The supplementary figure below depicts individual data points and shows data from excluded individuals.


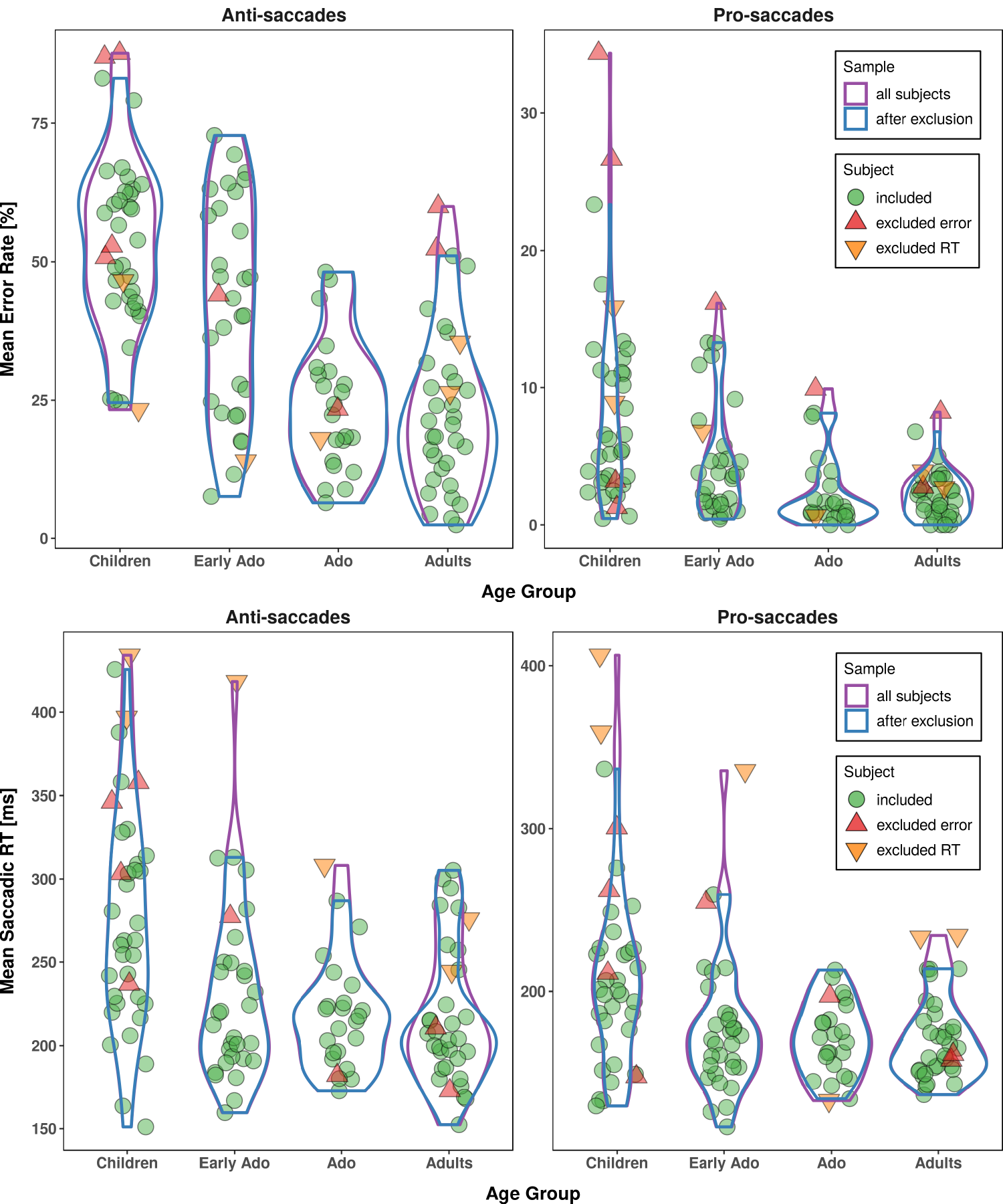


Supplementary figure 1: *Violin plots of global (i.e. across image categories) error rate and latencies for anti-saccades and pro-saccades separately when including all subjects (purple outline) in comparison to the reported analysis (blue outline). Upwards triangles indicate participants excluded because their error rate was more than 2SD away from the mean of their group. Downwards triangles indicate participants excluded because their reaction time was more than 2SD away from the mean of their group.*
